# The Association Between Ambient Temperatures and Hospital Admissions Due to Respiratory Diseases in the Capital City of Vietnam

**DOI:** 10.3389/fpubh.2022.903623

**Published:** 2022-07-19

**Authors:** Quynh Anh Tran, Vu Thuy Huong Le, Van Toan Ngo, Thi Hoan Le, Dung T. Phung, Jesse D. Berman, Huong Lien Thi Nguyen

**Affiliations:** ^1^Environmental Health Department, Hanoi Medical University School of Public Health, Hanoi, Vietnam; ^2^Division of Environmental Health Sciences, University of Minnesota School of Public Health, Minneapolis, MN, United States; ^3^Hanoi Medical University Hospital, Hanoi, Vietnam; ^4^Centre for Environment and Population Health, Griffith University, Southport, QLD, Australia; ^5^Ministry of Health (Vietnam), Hanoi, Vietnam

**Keywords:** temperature, hospital admission, respiratory disease (RD), cold and hot effects, Hanoi

## Abstract

This study aimed to examine the short-term effects of ambient temperature on hospital admissions due to respiratory diseases among Hanoi residents. We collected 34,653 hospital admissions for 365 days (November 1, 2017, to November 31, 2018) from two hospitals in Hanoi. A quasi-Poisson regression model with time series analysis was used to explore the temperature-health outcome relationship's overall pattern. The non-linear curve indicated the temperatures with the lowest risk range from 22 degrees (Celcius) to 25 degrees (Celcius). On average, cold temperatures showed a higher risk than hot temperatures across all genders and age groups. Hospital admissions risk was highest at 13 degrees (Celcius) (*RR* = 1.39; 95% *CI* = 1.26–1.54) for cold effects and at 33 degrees (Celcius) (*RR* = 1.21, 95% *CI* = 1.04–1.39) for the hot effects. Temporal pattern analysis showed that the most effect on respiratory diseases occurred at a lag of 0 days for hot effect and at a lag of 1 day for cold effect. The risk of changing temperature among women and people over 5 years old was higher than other groups. Our results suggest that the risk of respiratory admissions was greatest when the temperature was low. Public health prevention programs should be enhanced to improve public awareness about the health risks of temperature changes, especially respiratory diseases risked by low temperatures.

## Introduction

In recent years, substantial international literature indicates a link between ambient temperature changes and adverse health outcomes. The temperature–morbidity relationship has been found to be significantly associated with total and cause-specific morbidity in the short-term, especially in extremely high temperatures (1). Heat exposure is also reported to have an adverse effect on mortality in the elderly (2). However, a study in Hongkong found that cold temperatures contributed to a greater burden of death than hot temperatures (3). The previous studies have illustrated that cause-specific morbidity and mortality are attributable to cold and hot weather, such as diarrhea (4), cardiovascular diseases (5), and respiratory diseases. The inverse associations in which temperature decreases were associated with increases in cardiovascular and respiratory disease mortality were found in both places with desert climate and subtropical climate (6, 7). Temperature variability was reported as a risk factor for hospital admission for respiratory diseases among the elderly in Hongkong (8), and among women and young population in China (9).

Several studies were conducted in Vietnam to examine the associations between temperature, morbidity, and mortality. Most of these studies were conducted in Ho Chi Minh City, the most populous city in the country, and the Mekong Delta Regions (MDR), located in the South of Vietnam with the tropical weather condition. Therefore, these studies focus more on the heat effect than the cold effect. High temperatures were found to significantly impact hospitalizations related to infectious and respiratory diseases among the population at MDR (10). Extreme temperatures were also reported to increase the risk of cardiovascular admissions (11), respiratory disease admissions, and mortality in Ho Chi Minh City (12). Besides, studies conducted in a subtropical city in the Central region of Vietnam have suggested that the effects of high and low temperatures on hospitalization vary by climate zones (13, 14).

Respiratory diseases impose an immense worldwide health burden. Pneumonia and respiratory infections were ranked as two of the ten leading causes of morbidity in Vietnam and Hanoi region (15). According to the Vietnam climate change scenarios report, the country's average temperature has increased in recent decades, approximately 0.62 degrees Celsius (°C). Besides, the number of days with extremely low temperatures also increased in the North (16). However, little research has been done on temperature-related respiratory diseases in Hanoi, the capital and the second-most populous city in North Vietnam. Hanoi has warm and cold humid subtropical weather that is different from the tropical climate in the south. To date, there is one study that found a correlation between temperature, air pollutants concentration, and the number of hospital visits due to respiratory and cardiovascular diseases among children and the elderly (17). A another study reported ozone as a risk factor for respiratory admissions among children under 5 years old in Hanoi (18). These might not reflect the pattern of the relationship in other age groups.

This study aimed to examine the short-term effects of ambient temperatures on hospital admissions due to respiratory diseases among Hanoi residents.

## Materials and Methods

### Study Site

This study was conducted in Hanoi City, the capital of Vietnam, which is located in the Red River Delta region in the North. The total area of the city is 3,358.6 km^2^, such as 12 urban districts, one town, and 17 rural districts. Hanoi is the second-most populous city in Vietnam, with an average population of 7,520.7 thousand persons and a population density of 2,239 persons/km^2^ (19).

Hanoi has a subtropical climate with the annual average temperature ranging from 16.5°C in January to 29.1°C in July; monthly mean humidity ranges between 70 and 80% (16).

### Hospital Admission Data

The study outcome is daily hospital admission counts for respiratory diseases, which were obtained from medical records of two provincial hospitals in Hanoi, Vietnam, named St. Paul and Thanh Nhan hospitals (with the number of beds being 600 and 850, respectively). The data were collected from November 1, 2017 to November 31, 2018. All respiratory diseases (J00-J99) were selected for the study using ICD10 (20). We excluded all patients who were not living in Hanoi.

The medical data analyzed for this study did not involve individually identifiable health information, such as identification card numbers and health insurance codes. Informed consent was not specifically obtained. Each record has the dates of admission and discharge, types of diseases (based on International Classification of Diseases, Tenth Revision codes- ICD10), age and sex of patients, and city of residence.

### Weather Data

Weather data were provided by the Hanoi Environmental Protection Agency (Hanoi EPA) for the same period of hospital admission data, from November 1, 2017 to November 31, 2018. The data included daily average temperatures (°C), daily average relative humidity (RH) (%), the daily concentration of particulate matter (μg), and the daily concentration of ozone (μg).

### Statistical Analysis

We applied the times series analysis using a quasi-Poisson regression model to examine the risk of daily hospital admissions concerning changes in the temperatures (°C). The modeling framework can describe non-linear relationships both on the day of exposure and along with lags. We controlled for potential confounding effects of time-variant variables, such as the day of the week, holidays, seasonality trends, and daily RH.

Day of the week was used indicator variable described as Monday to Sunday. Holiday, which was defined as all Vietnamese public holidays (21), was a binary variable (holiday = 1, non-holiday = 0). To control for the seasonality trend, we adjusted for four seasons per year: Spring (Feb–Apr), Summer (May–Jul), Autumn (Aug–Oct), and Winter (Nov–Jan). Since hospitalization data were collected for one year only, it was not possible to control for a long-term trend in which temperatures may differ from year-to-year. Distributed lag non-linear model (DLNM) was applied to examine the non-linear and delayed effects of exposure on the outcome using natural cubic splines function with 4 degrees of freedom for temperature and lag days (11, 14). The delayed effects (lag effects) of temperature on respiratory diseases have been shown in literature and usually last for a week (22, 23). Therefore, we chose the lag time from 0 to 7 days. The median value of temperature was defined as the reference temperature for calculating relative risks (*RR*).

*Log E(Y*_*t*_*)*= δ + ε *(Temp*_0−7_*)* + ϕ*(Day)* + γ *(Holiday)* + ∂*(Season)* + μ*(RH)*

*where* Y_t_ is the number of hospital admissions on day t, δ is the intercept, ε is the regression coefficient for temperature, and *Temp*_0−7_ is the average daily temperature, and the lag days for 0–7 days; ϕ is the vector of coefficients for the day of the week, the *day* is the day of the week indicator variable on day t, γ is the regression coefficient for holiday status on day t, and *Holiday* is the public holidays. Controlling for seasonality trends, ∂ is the regression coefficient for season status on day t, and *Season* describes season variable with four different seasons per year (Spring, Summer, Autumn, and Winter). μ is coefficients for RH, and RH is the daily RH.

Sensitivity analysis was used to test the different model's choices. First, we changed the number of delayed effects (lag 0–5 days, lag 0–10 days, and lag 0–21 days) to test for the different delayed effects of temperature on hospital admissions due to respiratory diseases. Second, we adjusted by dual pollutant models, such as daily O_3_ and PM_2.5_ simultaneously. Third, we separated models with each air pollutants. Finally, we used a case-crossover study design, a special case of time series analysis, combined with DLNM to explore the exposure–outcome curves. Since we controlled for time and seasonal trends using two variables: day of week and season in the main model. Therefore, we used a time-stratified case-crossover study design to stratify by day of week and season using quasi-Poisson regression models.

## Results

Table 1 shows the summary statistics of daily hospital admissions counts and stratified by age and gender, and daily weather conditions. On average, the mean of daily hospital admissions counts was 95.16. The mean daily hospitalization count of people over 5 years of age was 60.98, which was nearly two times higher than that of people under and equal 5 years old (34.27). The mean daily count of men was higher than women (50.23 and 45.76, respectively). In terms of weather conditions, the daily mean temperature was 24.31°C, and the lowest and highest temperatures were 10.70 and 35.85°C, respectively.

**Table 1:** Summary statistics of daily hospital admissions counts and daily hospital admissions counts stratified by age and genders, and daily weather conditions.

**Variables**	**Mean (SD)**	**Minimum**	**25th**	**50th**	**75th**	**Maximum**
**Hospital admissions**	95.16 (42.21)	11	53	104	127	200
**Hospital admissions by age**
>=5	34.27 (12.26)	5	26	34	43	69
<5	60.98 (33.55)	3	23	71	87	139
**Hospital admissions by gender**
Female	45.76 (21.71)	6	26	49	64	104
Male	50.23 (21.98)	6	30	54	65	113
**Weather conditions**
Mean temperature (°C)	24.31 (5.37)	10.70	20.73	25.02	28.41	35.85
Relative humidity (%)	67.49 (13.90)	23.86	59.14	70.51	78.65	89.99
PM_2.5_ (mg/m^3^)	36.88 (7.91)	23.91	30.89	35.36	41.69	64.08
Ozone (mg/m^3^)	5.61 (11.21)	0.30	1.45	2.64	6.38	95.80

**Figure 1:**
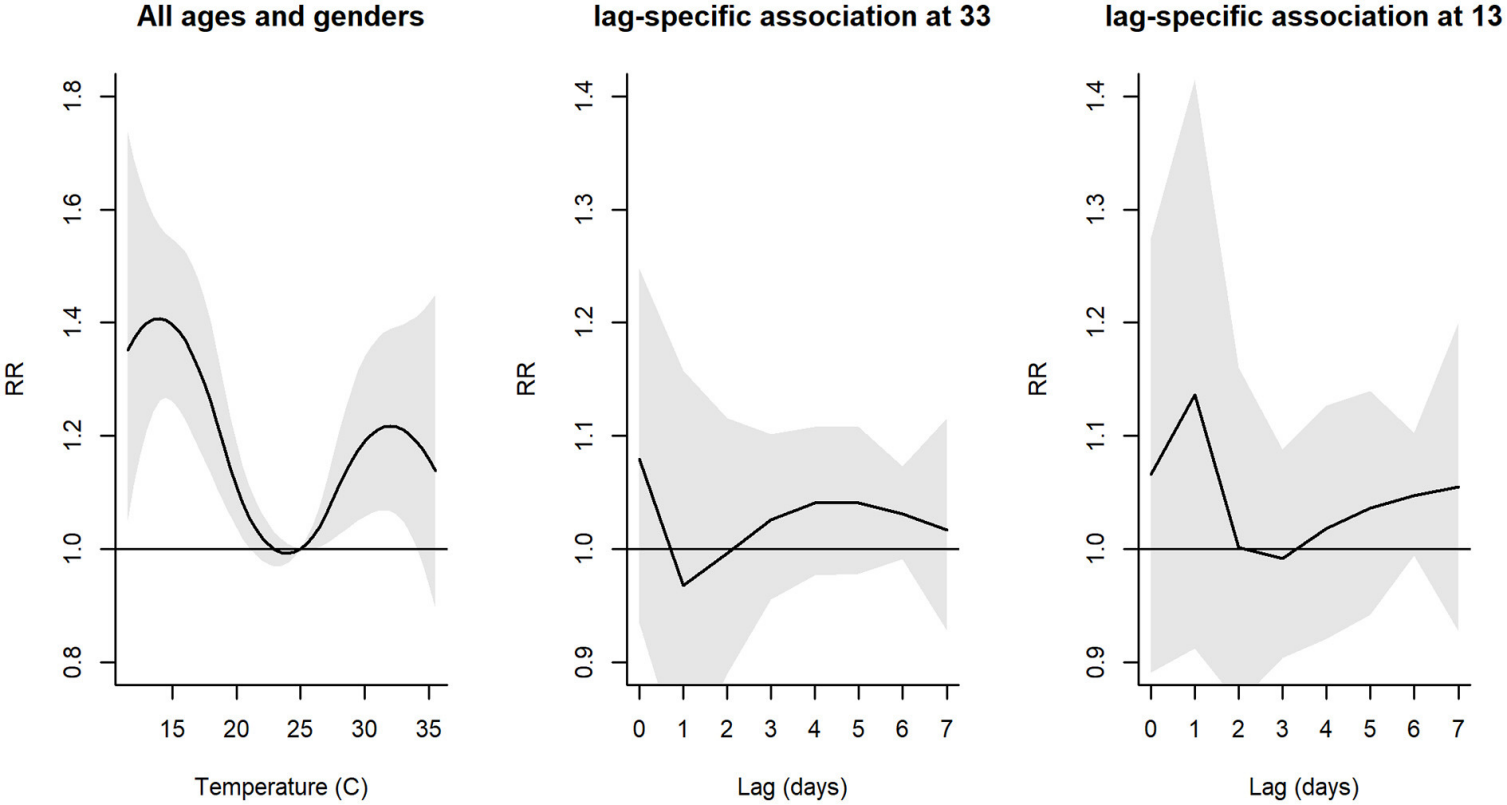
Plot of overall relative risk (RR) between daily mean temperature and hospital admissions, and individual lag-response curves for hot and cold effects. Shaded areas denote 95% confidence intervals (CI).

Slopes of overall exposure–response curves are summarized in Figure 1. The negative effects of temperatures on hospital admissions for respiratory diseases were observed at 22–25°C, with the lowest risk at 24°C.

The exposure–response curve reached the highest point at 13°C for cold temperatures (*RR* = 1.39, 95% *CI* = 1.26–1.54) and at 33°C for hot temperatures (*RR* = 1.21, 95% *CI* = 1.04–1.39). For hot effects, the delayed effect curves showed the highest effect at a temperature lag of 0 days; then the curve decreased on following days. For cold effects, the curves showed the highest effects at a temperature lag of 0 days and decreased at temperature lags of 1, 2, and 3 days, then increased after a temperature lag of 4 days.

**Figure 2:**
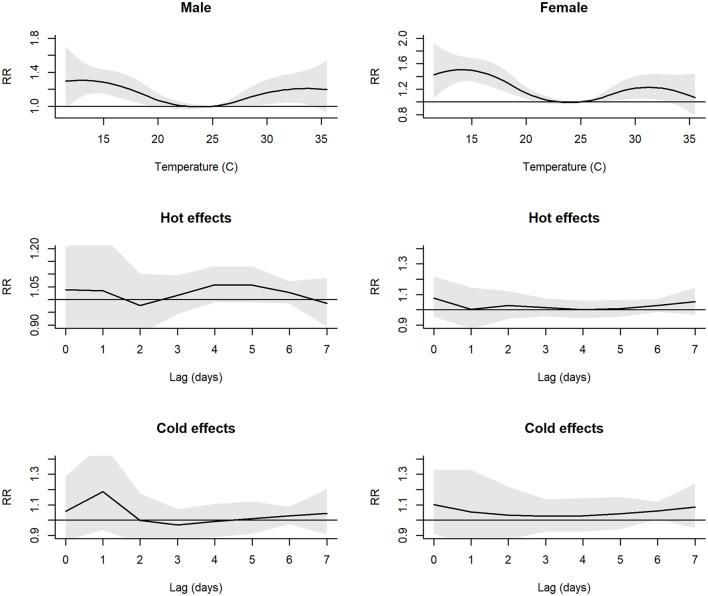
Plot of RR between daily mean temperature and hospital admissions and individual lag-response curves for hot and cold effects, stratified by genders. Shaded areas denote 95% CI.

Figure 2 reveals the non-linear association between the *RR* of hospital admissions and changes in mean daily temperature, stratified by genders and ages. We observed an increase in risk of respiratory diseases for cold and hot effects compared to a reference temperature of 25°C. For hot effects, the risk revealed increasing until about 32°C (*RR* = 1.20, 95% *CI* = 1.03–1.40) after which it remained constant among men, but among women, the risk increased, then decreased after 31°C (*RR* = 1.29, 95% *CI* = 1.05–1.43). The individual lag curves showed the greatest effects at a temperature lag of 0 days, except for cold effects for men.

**Figure 3:**
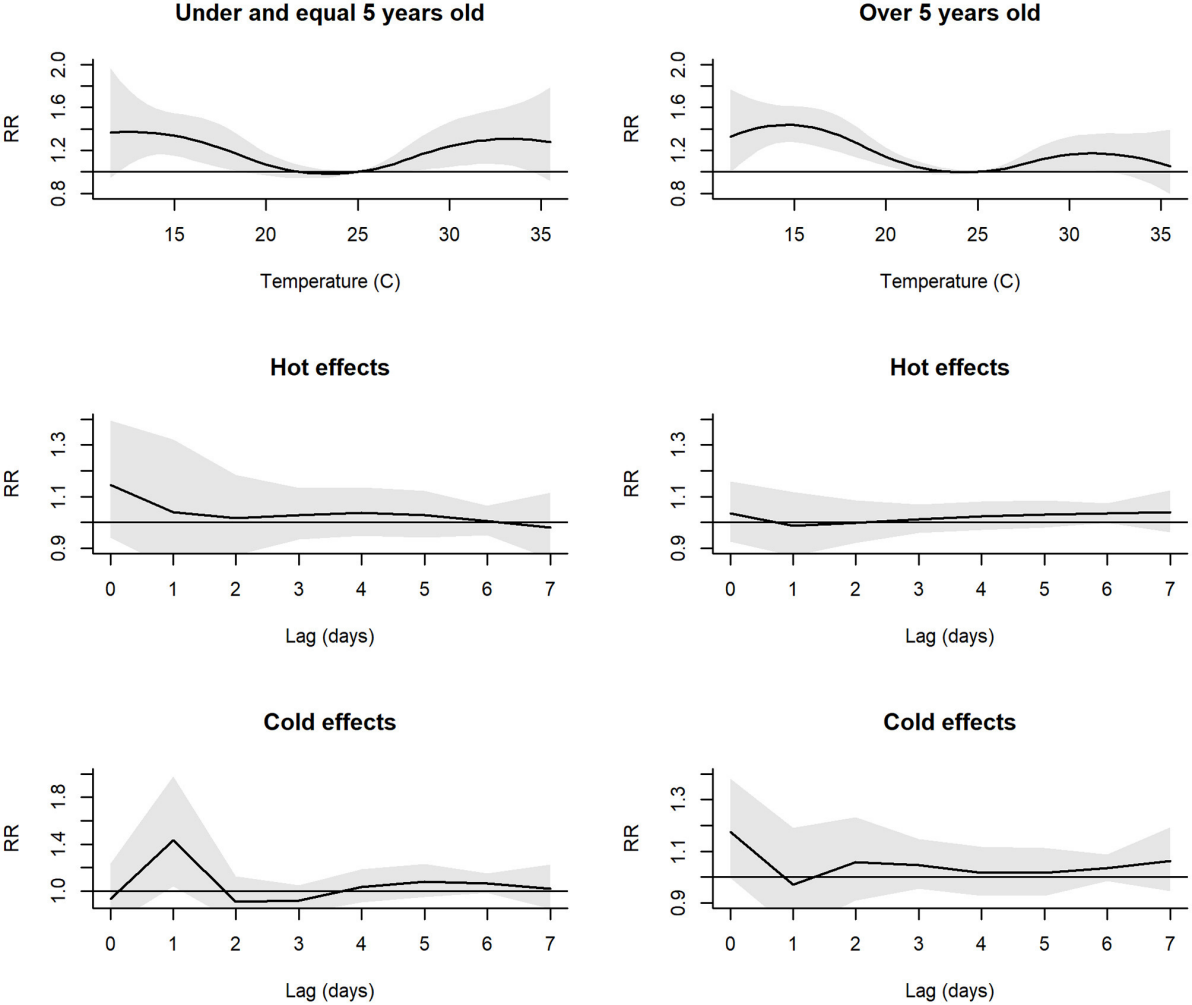
Plot of RR between daily mean temperature and hospital admissions and individual lag-response curves for hot and cold effects, stratified by age groups. Shaded areas denote 95% CI.

The risk of hospital admissions due to respiratory diseases differed between age groups (Figure 3). The greatest *RR* for cold effects of people over 5 years old (*RR* = 1.43, 95% *CI* = 1.26–1.71) was higher than that of people under and equal to 5 years old (*RR* = 1.33, 95% *CI* = 1.15–1.54). For hot effects, among people under and equal to 5 years old, the *RR* increased and remained at 33°C (*RR* = 1.30, 95% *CI* = 0.97–1.43). Meanwhile, among people over 5 years old, the hot effects increased, then reduced after 31°C (*RR* = 1.29, 95% *CI* = 0.84–1.43), and at the highest temperature (35°C), the *RR* was close to 1. The greatest risk of temperatures on hospital admissions was at a temperature lag of 0 days for hot effects. The greatest cold effects on people under and equal to 5 years old were at a temperature lag of 1 day.

Sensitivity analyses performed to assess the impact of model choices on our results. Changing the number of lag days from 0–7 days to 0–5 days and 0–10 days did not change the shape of the exposure–response curves. However, it did change the shape of the curve when the number of lag days was 0–21 (Supplementary Figure 1). Increasing the number of lag days may lead to overestimating our results at very cold and very hot temperatures. In addition, we included PM_2.5_ and O_3_, and we observed similar trends in the main models (Supplementary Figure 1). Finally, *RR* curves produced similar results in the sensitivity analyses, where a time-stratified-case-crossover study design was utilized (Supplementary Figure 2). Results were found to be robust against different choice of model parameters.

## Discussion

The findings of this study showed the same pattern as previous studies that both cold and hot temperatures could increase the risk of hospital admissions due to respiratory diseases (3, 4, 9, 10, 24). We observed that the highest risks occurred at a temperature of 13 and 33°C for cold and hot effects, respectively, while the low risk occurred when temperatures ranged from 22 to 25°C. These results suggest that temperatures below 22°C and above 25°C are associated with an elevated risk of hospital admissions due to respiratory diseases among the population in Hanoi, a subtropical capital city.

To our knowledge, this is the first paper to examine the relationship between temperatures and the cause-specific morbidity of respiratory diseases in Hanoi, the second most populous city in Vietnam. One previous study found that cold temperatures increased mortality among the older population in Hanoi with a higher risk for women but did not investigate the cause-specific mortality (25). Cold weather was also associated with cardiovascular hospital admissions among the elderly in a city in the North of Vietnam but not Hanoi (26). Some other published studies have been reported the link between temperatures, air pollution, and morbidity of respiratory and cardiovascular diseases in Hanoi but among children and the elderly population (17, 18).

Although both cold and hot temperatures have significant effects on hospital admissions due to the respiratory diseases, our results showed that cold temperatures have greater effects on hospital admissions than hot temperatures (*RR* = 1.39; 95% *CI* = 1.26–1.54 and *RR* = 1.21, 95% *CI* = 1.04–1.39, respectively). This is in line with the previous studies. For example, a study conducted in Hongkong reported that wintertime temperature was associated with a higher risk of incident respiratory diseases than summertime temperature (26), while another study found that cold temperature contributed to a higher mortality risk than hot temperature (3). In this study, we found that the minimum admission temperature was 24°C. This threshold is slightly higher than those reported in a study in Beijing, China that temperature of 21.5°C was the lowest risk of respiratory emergency department visits (24); and slightly lower than a threshold around 29°C for hospitalization of cardiovascular and respiratory diseases among the population living in the south tropical of Vietnam (11, 12). This could be explained by the difference in climate region. Beijing, China has a humid continental climate; Hanoi, Vietnam, has a humid subtropical climate. The south of Vietnam, like Ho Chi Minh City, has a humid tropical climate (according to Koppen Climate Classification). Another possible explanation is the human adaptation to the surrounding environment. People living in cold-weather regions would be able to adapt to hot weather less than those living in hot weather regions and vice versa.

Our results showed an increase in the risk of hospital admissions when the daily mean temperature increased above 25°C and decreased below 22°C, and the highest point was at 13 and 33°C. The risk reduced after those thresholds do not mean that extreme weather has lower risks, compared to non-extreme weather. In our study, the risks of temperatures above 33°C and below 13°C were higher than a reference temperature at 25°C. Future studies are warranted to determine the effects of extreme weather on hospitalization.

We have explored temporal patterns of the association between exposure to temperature over previous days and health outcomes on a particular day. In general, the previous studies indicated shorter lags during warmer seasons and longer lags during cooler seasons (1, 9). In this study, we found a lag of 0–1 days for cold effects and a lag of 0 day for hot effects. While the impact of high temperature on health among the Vietnamese population has been reported and less known about the effects of low temperature, our study found a significant contribution of cold impact on respiratory diseases hospital admissions among a sample population in the North Vietnam. The previous studies suggested that inhaling cool air, cooling the body surface, or cold stress causes pathophysiological responses that may contribute to increased susceptibility to respiratory infections (27). For example, cooling of the body surface may cause reflex vasoconstriction in the nose and upper airways, which may inhibit the respiratory defense (28). Besides, breathing cool air directly causes cooling of the upper respiratory tract and drying mucosal membrane, which can lead to epithelial damage in sensitive individuals (29). The study also indicated that cold temperature and low humidity play an important role in the occurrence of respiratory tract infections (30). Although Hanoi has hot and wet subtropical weather, the moisture in the wintertime is relatively low (minimum at 23%, as shown in Table 1), and the correlation between temperature and humidity is significantly positive (Supplementary Table 1). These can be possible explanations for the low temperature–respiratory hospitalization relationship in our study.

There are some limitations to this study. First, the hospital admissions data were collected from two provincial hospitals' medical records that may not reflect the admissions of the whole residents in Hanoi because hospital data might have been collected from other health service providers such as national or district hospitals. Second, data collected in a single year may not be strong to support stratified analysis, but the number of hospital admissions was large (total of 34,653). Therefore, it was suitable to apply ecological study and time series analysis. The short study period limited the precision of our estimates and our ability to adjust for a time variable because under, or over-smoothed estimations may occur based on different choices for degrees of freedom per year. Furthermore, for one year period, controlling for long-term trends in temperatures is not possible because this study did not collect year-to-year data. Last, the M shape was reported as the shape of temperature and hospital admissions, and the M shape is not robust at both ends because of the impact of outliers. We did not report the effects of the lowest and highest temperature as the main result.

Our study has several strengths. First, this study is the first study conducted in Hanoi to examine the short-term effects of ambient temperatures on hospital admissions due to respiratory diseases across all ages of Hanoi residents. Second, we performed sensitivity analysis to adjust the interactive effects of air pollution (PM_2.5_ and O_3_) on temperature-health outcome curves (Supplementary Figure 1). To our knowledge, this technique has not been tested in the previous studies on temperature-related health outcomes in Vietnam (10, 12, 13, 25), while it has been presented in a recent study in the USA (31). Therefore, our study may be the first study using air pollutants as factors to control for the association between temperatures and hospital admissions due to respiratory diseases in Vietnam. We did not include air pollution in the main model because the measurement of air pollution in Hanoi was not comprehensive. All air quality stations were in urban Hanoi and lacked in rural Hanoi. If we included air pollution in the model, we might be faced with overestimation or underestimation because of information bias.

In conclusion, our study is the first study to examine the short-term effects of temperatures on hospital admissions due to respiratory diseases among only Hanoi residents. Our results represent a temporally robust investigation and support the hypothesis that low temperature could be an important risk of respiratory diseases. The findings of this study, conducted in a subtropical and populous city of Vietnam, consolidated the results of the previous studies that both cold and hot weather had impacts on respiratory disease morbidity across all ages in subtropical regions. As Vietnam is acknowledged as one of the world's most vulnerable countries to the effects of climate change, including the rise of temperatures and the occurrence of extremely low temperatures in the North, prevention programs should be enhanced to improve public awareness about the health risks of temperature changes and the importance of reducing climate change causes.

## Data Availability Statement

Providers do not allow to generate the dataset. Requests to access these datasets should be directed to le000156@umn.edu, VL.

## Ethics Statement

The studies involving human participants were reviewed and approved by Hanoi Medical University. Written informed consent for participation was not provided by the participants' legal guardians/next of kin because: This is the secondary data, which did not collect data on human, we collected on health records database.

## Author Contributions

QT: collecting data. QT and VL: writing different part of the original draft and analysis. VN, TL, VL, and HN: editing. DP, VL, QT, and JB: conceptualization and methodology. VN, TL, VL, DP, JB, and HN: review. All authors contributed to the article and approved the submitted version.

## Conflict of Interest

The authors declare that the research was conducted in the absence of any commercial or financial relationships that could be construed as a potential conflict of interest.

## Publisher's Note

All claims expressed in this article are solely those of the authors and do not necessarily represent those of their affiliated organizations, or those of the publisher, the editors and the reviewers. Any product that may be evaluated in this article, or claim that may be made by its manufacturer, is not guaranteed or endorsed by the publisher.
