# Supplementary material for: The Association Between Ambient Temperatures and Hospital Admissions Due to Respiratory Diseases in the Capital City of Vietnam
Source: Front Public Health. 2022 Jul 19;10:903623. doi: 10.3389/fpubh.2022.903623 (PMC9350518; doi:10.3389/fpubh.2022.903623)
Supplement: Supplementary file 1 [file Data_Sheet_1.docx]

|  | Ozone | Temperature | RH | PM_2.5_ | HA |
| --- | --- | --- | --- | --- | --- |
| Ozone | 1 |  |  |  |  |
| Temperature | 0.06 | 1 |  |  |  |
| RH | -0.03 | 0.22* | 1 |  |  |
| PM_2.5_ | 0.09 | -0.46* | -0.05 | 1 |  |
| HA | 0.03 | 0.01 | 0.01 | 0.06 | 1 |

*: p<0.05

***Supplement Table 1:*** *Correlation of hospital admissions, ozone, temperature, relative humidity and PM_2.5_*

Supplement 1 indicates the correlation of metrics and pair p value. On average, daily hospital admissions showed the weak positive correlation with daily mean temperature (0.01), ozone (0.03), relative humidity (0.01), and PM_2.5_ (0.06) but no significance difference was observed. Additionally, the correlation between daily mean temperature and PM_2.5_ was negative (-0.46) with significant difference.


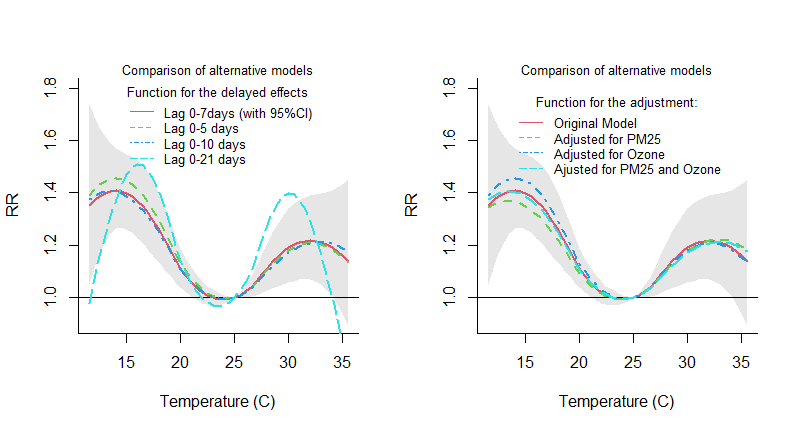


***Supplement Figure 1***: Plot of the comparison of alternative models of different lag days (left side) and models adjusted by daily ozone, daily PM_2.5,_ and the combination of daily ozone and daily PM_2.5_ (right side)


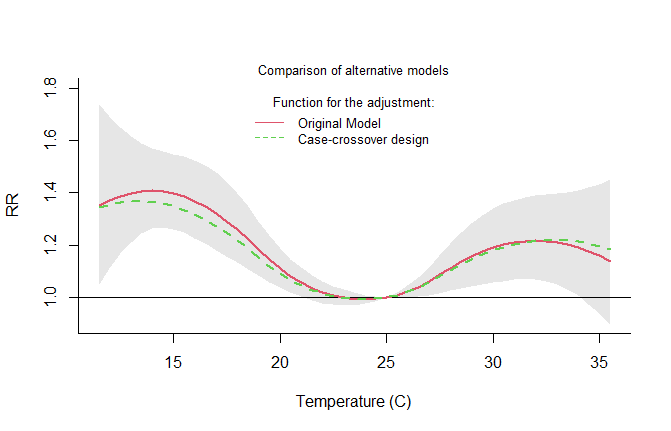


***Supplement Figure 2***: Plot of the comparison of an alternative model using case-crossover study design approach
